# 

**Published:** 1881-06

**Authors:** 


					﻿Whole Number,
CCXXXxIX.
JUNE, 1881.
Number ii,
Volume XX.
THE
BUFFALO
Medical and Surgical Journal.
EDITORS:
THOS. LOTHROP, M.
f». W. VAN PEVMA, M. !>.,
A. R. DAVIDSON, M.
ULTCIK1N HOWE, M.
HERMAN MYNTER, M. I>.
BUFFALO:
Published by the Medical Journal Association.
Read the Notice of this Journal among the Advertisements.
Entered at the Post-Office at Buffalo, N. Y., as Second-Class Mail Matter.
				

